# Correction of Disease Phenotype in Pompe Disease Knockout Mice Following Cationic Lipid-GL-67-Mediated Gene Therapy

**DOI:** 10.1089/dcbr.2025.0025

**Published:** 2025-05-09

**Authors:** Frank Martiniuk, Justin Martiniuk, Adra Mack, Seng H. Cheng

**Affiliations:** ^1^ JME Group Associates, Inc, Roseland, New Jersey, USA.; ^2^ PsychoGenetics Center, Paramus, New Jersey, USA.; ^3^ Alexion. AstraZeneca Rare Diseases, Boston, Massachusetts, USA

**Keywords:** lipid, GL-67, acid maltase deficiency, gene therapy, Pompe disease

## Abstract

**Background:**

Genetic deficiency of lysosomal acid α-glucosidase or acid maltase (GAA) results in Pompe disease (PD) or glycogen storage disease type II, encompassing subtypes of varying severity. The infantile form presents as hypotonia, muscle weakness, and congestive heart failure in the first year, while the adult onset forms are limited to skeletal muscle. Gene therapy for diseases that affect muscles or organs (as PD) may be very difficult due to their size or location and our ability to transfect these organs with high efficiency.

**Methods:**

We evaluated a plasmid vector carrying the cytomegalovirus promoter linked to the human *GAA* cDNA complexed with the cationic lipid GL-67 as a delivery system for therapy of PD.

**Results:**

A human PD fibroblast cell line was transiently transfected with increasing amounts of the cationic lipid GL-67 complexed to the human *GAA* expression plasmid. Cells showed a significant increase in human GAA activity with 20 nmol of the cationic lipid, resulting in maximal expression. GAA knockout mice were treated intraperitoneally every 2 weeks for four months with 100 µg of plasmid and 2 mM GL-67 lipid. Tissues, including heart, diaphragm, and skeletal muscle showed substantial increase in GAA activity. More important, the clinical phenotype of hind and forelimb muscle weakness was reversed and life span extended to almost normal longevity.

**Conclusions:**

These data suggest that cationic lipid mediated gene delivery of the human *GAA* cDNA may be an effective treatment for PD.

## Introduction

Acid α-glucosidase or acid maltase (GAA) is a lysosomal enzyme that primarily hydrolyzes linear α 1-4 glucosidic linkage ranging from large polymers (glycogen and amylose) to maltose.^1,2^ The enzyme is synthesized and processed via a pathway common to lysosomal enzymes.^3,4^ GAA is a “house-keeping” enzyme with all tissues and cell types exhibiting some activity.^5^

Pompe disease (PD) or acid maltase deficiency (AMD) results in a spectrum of at least five clinical phenotypes including an infantile disorder (IOPD),^6,7^ a nonclassical infantile form, a childhood-juvenile, and a late-onset adult myopathy.^8,9^ The clinical phenotype can be defined by age of onset, severity of muscle dysfunction, and tissue involvement. IOPD has essentially complete enzyme deficiency in all tissues and massive accumulation of glycogen in skeletal and heart muscle. Death usually occurs within the first year of life as a result of severe cardiomyopathy and respiratory failure. The nonclassical infantile form presents with less severe cardiomyopathy and absence of left ventricular flow. Longer survival can be achieved with assisted ventilation and supplemental nutrition.^10^ The later onset forms (LOPD), childhood, juvenile, and adult, have residual enzyme activity in most tissues with varying amounts of glycogen accumulation, primarily in skeletal muscle^5,9^ and only exhibit progressive skeletal muscle weakness and wasting. The incidence of PD is ∼1/18,000 births or lower^11–14^ (Orpha number-ORPHA365 at www.orpha.net) and based on this incidence, there are ∼8,000 accumulated cases in the United States and >300,000 cases worldwide.

Currently, there is no cure for PD. Enzyme and gene replacement therapies (GT) are in development. Enzyme replacement therapy (ERT) by Sanofi/Genzyme, Inc. with alglucosidase alfa or Myozyme/Lumizyme—a recombinant human acid α-glucosidase secreted from CHO cells, has shown moderate success using a biweekly infusion regimen. In August 2021, they received FDA approval for Nexviazyme (avalglucosidase alfa-ngpt) for treating late-onset PD (LOPD) patients aged 1 year and older. FDA recently (September 28, 2023) approved edible Pombiliti (cipaglucosidase alfa-atga) and Opfolda (miglustat) for LOPD not improving on ERT.

We have previously cloned and sequenced the authentic functional cDNA for human *GAA*^15,16^ and determined extensive genetic heterogeneity among patients as detected by gross abnormalities of mRNA and DNA. Approximately half of infantile onset patients lack *GAA* mRNA, while many adult onset patients exhibit mRNA of altered size and/or amounts.^15–18^ In total, there are at least 422 confirmed disease specific mutations at the *GAA* locus^19–23^ and the promoter region and enhancer segments have been identified^24–29^ (www.pompevariantdatabase.nl).^29,30^

Naturally occurring animal models of PD have been identified.^31–35^ At least four mouse models have been developed.^36,37^ Raben et al.^36^ generated a GAA knockout (KO) mouse that lacks GAA activity and recapitulates the critical features of the IOPD and LOPD forms. This mouse (exon 6^neo^, Jackson Labs (B6;129-Gaa^tm1Rabn^/J) accumulates glycogen in the lysosomes of cardiac and skeletal muscles including the diaphragm by 3 weeks, plus develops muscle wasting, reduced mobility, and strength by 8–9 months. When these mice were treated with a rhGAA (20–100 mg/kg/week), skeletal muscle took up little enzyme compared with liver and heart with glycogen reduction.^38^ Their second mouse has a deletion of exon 6 and unimpaired muscle strength but is identical to the exon 6^neo^ disrupted mouse with respect to biochemical and pathologic parameters. Raben et al.^39,40^ provided evidence for the role of autophagy in an autophagic-free GAA KO mouse (*Atg*7 KO).^41–44^ Douillard-Guilloux et al.^45,46^ in a GAA/glycogen synthase 1 KO mouse, exhibited a reduction of glycogen in heart and skeletal muscles, a decrease in lysosomal autophagy and correction of cardiomegaly, demonstrating long-term elimination of muscle glycogen synthesis leads to a significant improvement of metabolic defects. Kan et al.^47^ used CRISPR gene-editing to generate a homozygous c.1935C>A PD mouse that recapitulates IOPD.^48–52^ Huang et al.^53^ used CRISPR-Cas9 to generate a PD model that exhibits early-onset hypertrophic cardiomyopathy and skeletal muscle weakness. Munoz et al.^54^ developed an IOPD rat model with muscle-directed AAV GT.

Currently many attempts are being made to use gene therapy (GT) to treat inherited genetic disorders. Most of these methods have used replication defective retroviruses or adenoviruses directed at organs or cell types that can be infected at an efficient rate.^55,56^ Dunbar et al.^57^ reviewed the current status of GT with viral vectors, adoptive transfer of genetically engineered T cells, hematopoietic stem cells, and CRISPR gene editing. Nonviral GT and their products should reduce cost and minimize immune responses and toxicity. Approved GTs range from $375 K to $2–3 M. Excessive regulatory oversight creates an elongated and expensive route to approval.^58–64^

The Journal of Gene Medicine’s Clinical Trial site (www.abedia.com/wiley) is the most comprehensive source on worldwide GT clinical trials. In 2021, there were 3,180 trials with 1,820 USA and 482 were plasmid DNA. Viral GTs offer great rewards to treat many diseases but there are also significant risks including inflammation, immunogenicity forcing only a single administration even though a single administration may be insufficient to resolve chronic conditions and there are significant risks of off-target development of cancer or vector infusion reactions.^65–70^ CRISPR gene editing can have off-target effects and rearrange chromosomes and whether it can trigger cancer may not be known for several years. Compared with replication defective viruses, nonviral GT offers fewer safety concerns and, if recent studies are validated, has a wider range of cells. At least 8% of trials use nonviral delivery.^71,72^

There are many studies in PD cells and animal models using AV, AAV, lentivirus, and retroviruses.^73–97^ Barry Byrne and his colleagues at the Univ. of Florida and investigators at Duke University are developing GT using AAV systems.^76,93,94,98–113^ Additionally, 2 Phase I clinical trials of AAV mediated liver depot GT have also been initiated.^107^ Liver-specific expression of GAA established secretion from the liver accompanied by receptor-mediated uptake of GAA by the cation-independent CI-MPR corrected GAA deficiency and cleared the majority of glycogen in the heart/skeletal muscle, brain, plus induction of immune tolerance and reduced GAA antibodies.^107–113^ Eggers et al.^114^ showed that AAV-GAA delivery to muscle is efficacious in GAA KO mice.

Promising nonviral delivery systems are currently undergoing clinical trials.^115–126^ Successful GT rely on methods that safely introduce DNA into target cells and enable subsequent expression of proteins. Peptide DNA delivery facilitate condensation into nanoparticles, delivery into cells, and subcellular release to enable protein expression. Peptides are programable that can be designed to address biocompatibility, stability, and subcellular barriers that limit efficiency of nonviral GT systems. Recent peptide technologies utilize multidimensional structures, non-natural chemistries, and combinations of peptides with lipids to achieve desired properties and efficient transfection.^124,125^ Conventional nonviral methods are hydrodynamic pressure techniques, electroporation, ballistic bombardment, and microinjection. Cationic polymer methods include lipofection, cationic peptides, PEI, and receptor-mediated.

Lifelong ERT can be prohibitively expensive ($250,000–$650,000/adult/year) resulting in the reluctance of insurance companies to reimburse costs for LOPD,^127^ thus underlining the demand for more economical production and novel delivery strategies. Nonviral GT should reduce cost and minimize immune responses and toxicity. It has been generally accepted that the therapeutic threshold may be 10–30% of normal GAA would be required to reverse the myopathy/cardiomyopathy.^15,128–133^ Besides delivering the *hGAA* gene to tissues, transduced tissues might result in cellular “enzyme-factories” with the rhGAA secreted into the circulation and taken up by distant tissues. Compared with replication defective viruses, nonviral GT offers fewer safety concerns and, if recent studies are validated, has a wider range of cells.

Direct microinjection of DNA or RNA into muscles and expression of the genes has also been demonstrated.^134^ However, only a small number of cells are affected, which would not be applicable to PD. Increasing evidence indicates that cationic lipids are capable of transferring foreign genes *in vitro* and *in vivo* including DNA, RNA, and proteins. Potential advantages of cationic lipid–DNA complexes are that the DNA is not directly derived from replication competent virus and thus minimizes public health and vector safety concerns. Animal studies have revealed minimal toxicity from many cationic lipid formulations.^135–137^ Several cationic lipid combinations have been used as gene transfer vectors to deliver plasmid DNA *in vitro*, although some of these appear to show considerably enhanced efficacy in reporter gene assays in certain cell types. Many formulations have been studied in *in vivo* settings to improve the transfection efficiency and eliminate any toxic effects.^138–144^ A cationic lipid formulation GL-67/DOPE/DMPE-Peg5000 (1:2:0.05) reportedly has higher transfection efficiency *in vivo*.^145–149^ Gene therapy clinical trials using a variety of liposome formulations have been initiated for cancer, including brain, breast, colorectal, lung, melanoma, and renal tumors, and some monogenic diseases.^140–149^ In this report, we evaluated the parenteral administration of the cationic lipid GL-67 complexed with plasmid DNA in GAA KO mice for nonviral GT of PD.

## Materials and Methods

### Animal approval

The Institutional Animal Care and Use Committee at NYU School of Medicine protocols 030806, 050812, New York, NY USA.

### Subcloning the human GAA cDNA into expression vector-pcDNA3

The functional human *GAA* complete coding region cDNA (bp 1 to 3,150, 2,856 bp of coding cDNA) was subcloned into the EcoRI site of the pcDNA3 expression plasmid (Invitrogen, CA) driven by the cytomegalovirus (CMV) immediate early promoter-enhancer element. The DNA was prepared for transfection by PEG precipitation,^150^ followed by an additional phenol-chloroform extract/ethanol precipitation.

### Cationic lipid-mediated transient transfection

In six-well plates, plasmid DNA (pcDNA3 × human *GAA* cDNA) (10 µg) was transfected into 0.5 × 10^6^ TR4912 cells, an Ad5-SV40 immortalized PD fibroblast cell line (expresses no *GAA* mRNA and activity) with varying amounts (4–200 nmol) of the cationic lipid (GL-67) (Genzyme Corporation, Framingham, MA). The cells were harvested with a cell scraper 48 h later and lysed in 300 µL of 10 mM sodium phosphate (pH 7.5) by sonication. The lysate (50 µL) was assayed for GAA activity in 100 µL of the artificial substrate 4-methylumbelliferyl-α-D-glucoside (4-MU-Glyc) (1 mg/mL) in 0.5 M sodium acetate pH 4.0 for 2–6 h at 37°C and fluorescence was determined using a Sequoia-Turner fluorometer (excitation: 360_nm_ and emission: 460_nm_) (Sequoia-Turner, Mountainview, CA). For the assay of neutral α-glucosidase (NAG) as an internal control, 4-MU-Glyc in 0.5 M sodium phosphate at pH 7.5 was used.

### Parenteral delivery of cationic lipid-pcDNA × human GAA cDNA complexes into GAA KO Mice

GAA KO mice were treated intraperitoneally (IP) with 100 µg of pcDNA3 × human *GAA* cDNA plasmid and 2 mM of the cationic lipid GL-67 in a final volume of 100 µL with PBS. The GAA KO mice^36^ were sacrificed at various times and tissues removed and stored at −80°C. Motor activity was measured with a running wheel for 12/12 light/dark hours (Mini-Mitter Inc., Bend, OR) and fore-limb grip strength with a grip strength meter (Columbus Instr., Columbus, OH).

#### Polymerase chain reaction for human GAA plasmid

Polymerase chain reaction (PCR) conditions utilized Taq polymerase (Promega Inc, Madison, WI) according to instructions with 0.5 µg DNA from tissues or 1 ng of control pcDNA3 × human *GAA* cDNA. The human *GAA* cDNA primers for the outer set are:

5′-TGATGCTCAGCACCAGCTGGACCAGGAT-3′ (bp 800–827) and 5′-GAGCTGCTCCCAGGAGCTCCACACGT-3′ (actual sequence; bp 1872–1847). Nested primers are: 5′-CCGGTGCGAACCTCTACGGGTCT-3′ (bp 860–882) and 5′-CTTCGGTCAGGCCGTAGAGGTTGTGCAGGT-3′ (actual sequence; bp 1738–1709). The cycling conditions were: Disassociation at 94°C for 1.5 min, annealing at 67°C for 1.5 min, and extension at 72°C for 5 min, for 30 cycles, with the last cycle for 10 min. Nested PCR used 69°C for annealing. The PCR amplicons (20 µL) were electrophoresed in 1% agarose and visualized under UV light with ethidium bromide staining.

### Biostatistics

Student’s *t* test was used for comparisons of treated versus mock-treated GAA KO mice and a *p* ≤ 0.05 for statistical significance.

## Results

### *In vitro* studies

We transfected a PD fibroblast cell line (TR4912) using the cationic lipid GL-67 complexed to the functional pcDNA3 × human *GAA* cDNA. To optimize transfection efficiency and expression, we tested increasing amounts of GL-67. After 48 h, cells were harvested and assayed for GAA activity and as an internal control, NAG. Transfection with 20 nmol of GL-67 resulted in maximum expression of GAA in the cell lysates (Fig. 1).

**FIG. 1.**
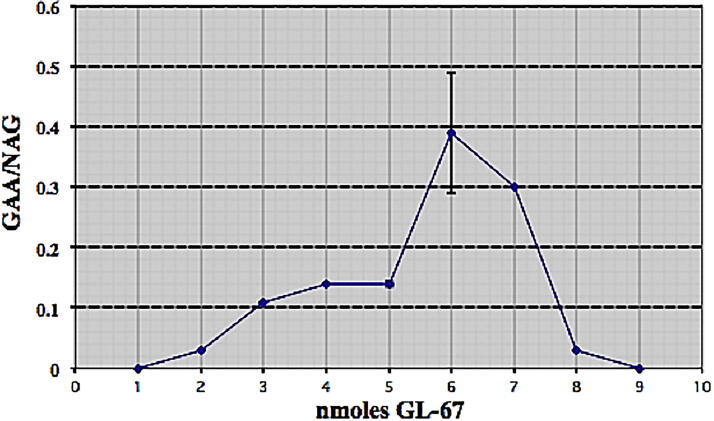
Transfection of cells with varying amounts of GL-67. Transfection of PD fibroblast cells (TR4912) using GL-67 with the pcDNA3 × human GAA cDNA minigene and GAA/NAG measured after 48 h. Maximal expression was at 20 mM GL-67 (mean + SEM, n = 3). PD, Pompe disease; GAA, acid α-glucosidase; NAG, neutral α-glucosidase.

### *In vivo* studies in GAA KO mice

In unpublished preliminary studies in normal mice, we investigated liposome-mediated gene transfer with the pcDNA3 × human *GAA* cDNA plasmid. A single IP treatment resulted in plasmid uptake and persistence in all tissues, including heart and skeletal muscle for at least 90 days. Multiple treatments increased plasmid content and longevity of expression. Human GAA activity was found in all tissues (data not shown).

Because the data in normal mice suggested that a multiple treatment regimen resulted in widespread GAA expression, we initiated experiments in GAA KO mice with exon 6^neo^ insertion.^36^ We parenterally administered GAA KO mice using 2 mM lipid GL-67 and 100 µg of pcDNA3 × human *GAA* cDNA in 100 µL PBS. Mock-treated GAA KO mice received the vector containing no human *GAA* cDNA and normal mice (strain 129j) received saline. Animals (n = 3 in each group) were sacrificed at 1 month (two infusions) and DNA extracted from the various tissues. We analyzed for plasmid content by PCR and assayed for GAA activity. PCR for the human *GAA* cDNA showed that the amplicon was found in varying amounts in all tissues including lung, liver, skeletal muscle, and heart (Fig. 2). Treated GAA KO mice showed significant increase in GAA/NAG ratio ranging from 7% to 54% of normal (Table 1) (*p* mock-treated vs. treated were all ≤0.02 except kidney = 0.08). GAA activity in heart and diaphragm increased to one third of normal, while skeletal muscle GAA activity was 7% of normal. Lung, liver, and kidney had GAA activity ranging from 33% to 54% of normal.

**FIG. 2.**
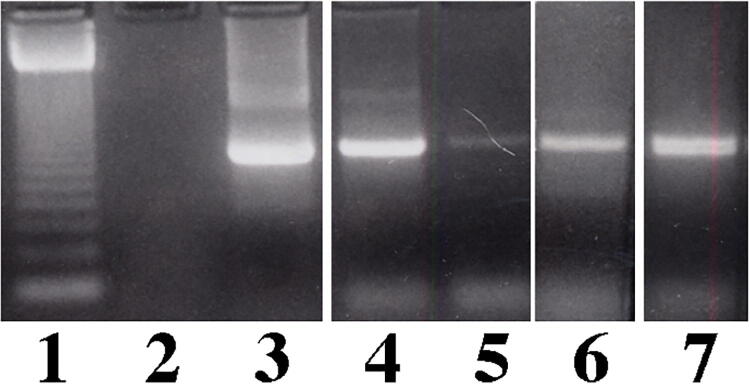
Electrophoresis of PCR amplicons from GAA KO mice. Agarose gel electrophoresis of PCR amplicons from GAA KO tissues for human GAA 30 days after administration of two IP infusions (14 days apart) of GL-67 and pcDNA3 x human GAA cDNA. Lane 1: 123 bp DNA standard, lane 2: Normal mouse 129 strain, lane 3: Plasmid pcDNA3 × human GAA cDNA, lane 4: GAA KO mouse treated with lipid GL-67 and human GAA minigene from heart, lane 5: liver, lane 6: skeletal muscle, and lane 7: lung. PCR, polymerase chain reaction; KO, knockout; IP, intraperitoneal.

**Table 1.** GAA/NAG in normal, mock-treated, and treated GAA KO Mice

*GAA/NAG* mean (n = 3)								
	Skeletal muscle	Heart	Diaphragm	Liver	Lung	Kidney	Spleen	Brain
Mock-treated								
GAA KO mice	0.007 ± 0.002	0.042 ± 0.003	0.045 ± 0.005	0.075 ± 0.01	0.039 ± 0.006	0.19 ± 0.04	0.005 ± 0.002	0.05 ± 0.02
Normal mice	1.67 ± 0.1	0.56 ± 0.05	0.78 ± 0.09	0.9 ± 0.2	0.44 ± 0.1	0.67 ± 0.08	0.78 ± 0.09	1.6 ± 0.1
Treated GAA KO mice	0.10 ± 0.03	0.17 ± 0.04	0.28 ± 0.04	0.60 ± 0.3	0.20 ± 0.08	0.24 ± 0.04	0.13 ± 0.03	0.80 ± 0.1
(% of normal)	7%	35%	33%	54%	40%	33%	21%	42%

*p* mock-treated vs. treated = all ≤0.02 except kidney = 0.08.

GAA, acid α-glucosidase; NAG, neutral α-glucosidase.

To determine whether we could correct the disease phenotype of hind and forelimb muscle weakness in the GAA KO mice, we treated mice (3-4 months old) every 2 weeks for 4 months. Hind limb motor activity was measured by monitoring running wheel activity over a period of 18 h, while forelimb muscle strength was determined with a grip strength meter at release. After 1 month or three treatments, we observed an improvement in hind limb motor activity (Fig. 3). By the second month, hind limb motor activity was equal to age-matched control 129j mice. Treated GAA KO mice at second, third, and fourth months ran a significantly greater number of revolutions than the mock-treated GAA KO mice. Combining the data from months 2–4, mock-treated GAA KO mice (n = 3) ran a mean of 673 ± 52 (standard error of the mean; SEM) revolutions in 18 h and normal mice (strain 129j, n = 4) ran a mean of 5,323 ± 704 revolutions in 18 h. Treated GAA KO mice (n = 4) ran a mean of 4,669 ± 1,343 revolutions in 18 h (p ≤ 0.01 treated vs. mock-treated GAA KO mice). Simultaneously, we measured forelimb grip strength. Some improvement in forelimb strength was observed by 1 month. By 2 months, treated GAA KO mice had regained most of their forelimb grip strength, which was maintained for the entire 4 months (Fig. 4). Combining the data from months 2–4, normal mice (strain 129j, n = 4) averaged 235 ± 11 lbs. (SEM) grip at release. Mock-treated GAA KO mice (n = 3) averaged 85 ± 11 lbs. grip at release and treated GAA KO mice (n = 4) averaged 210 ± 9 lbs. grip at release (*p* < 0.001 treated vs. mock-treated GAA KO mice).

**FIG. 3.**
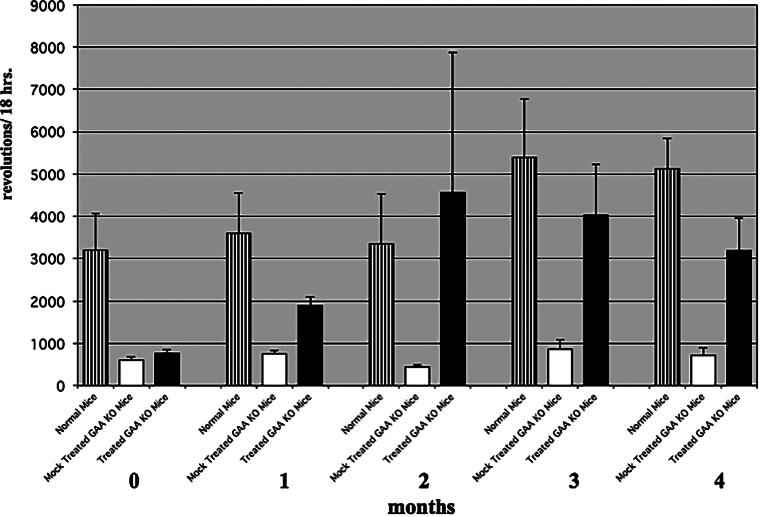
Running wheel activity. Running wheel motor activity of treated (clear) and mock-treated (black) GAA KO, plus normal (hatched) mice (strain 129j). GAA KO mice (3–4 months old) were treated every 2 weeks for 4 months and motor activity was evaluated in a free running wheel for 18 h and a 12:12 L/D photoperiod. Combining the data for months 2–4, the mock-treated GAA KO mice (n = 3) ran a mean of 673 + 52 revolutions in 18 h. Normal mice (strain 129j, n = 4) ran a mean of 5,323 + 704 revolutions in 18 h. Treated GAA KO mice (n = 4) ran 4,669 + 1,343 revolutions in 18 h (*p* < 0.01 mock-treated vs. treated).

**FIG. 4.**
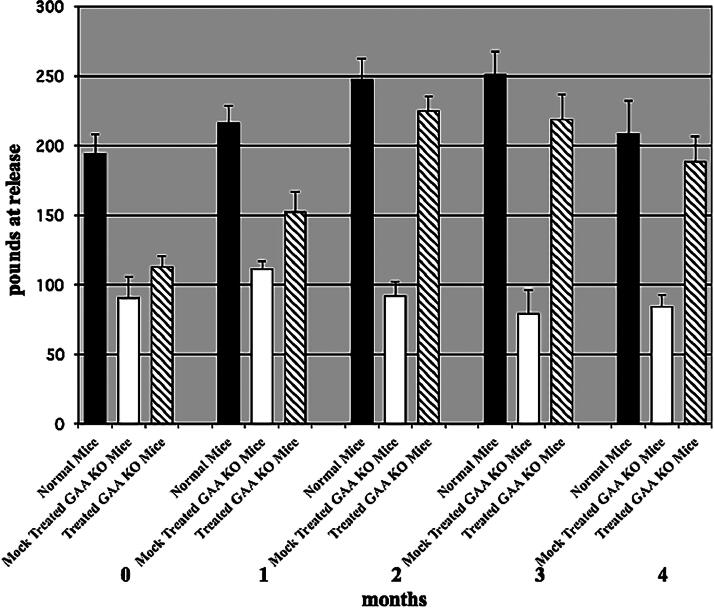
Forelimb grip strength. Forelimb muscle strength was measured by grip strength at release in normal, mock-treated, and treated GAA KO mice. Combining the data for months 2–4, normal mice (strain 129j, n = 4) averaged 235 + 11 lbs. (SEM) at grip release, mock-treated GAA KO mice (n = 3) averaged 85 + 11 lbs. at grip release, and treated GAA KO mice (n = 4) averaged 210 + 9 lbs. at grip release (n = 12) (p ≤ 0.01 mock-treated vs. treated). SEM, standard error of the mean.

Figure 5 shows the mean-SEM life span in months for normal-129j mice, mock-treated GAA KO mice, and different regimens of GL-67 mediated nonviral plasmid delivery to GAA KO mice. Normal 129j mice averaged 26 months survival (n = 12) while mock treated GAA KO mice averaged 9.6 months (n = 14). GAA KO mice treated by various protocols with GL-67 nonviral plasmid delivery of the pcDNA3 × human *GAA* cDNA plasmid showed 15–22 months survival—all statistically significant (p ≤ 0.01 to 3.0E-10) versus mock treated mice.

**FIG. 5.**
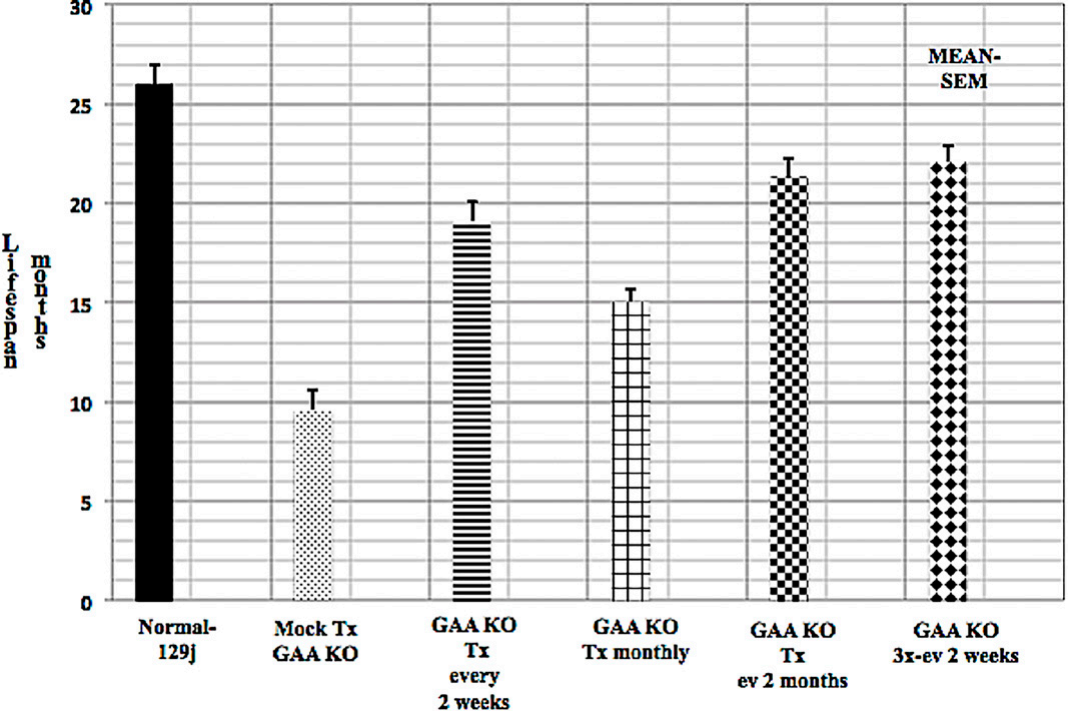
Life Span. Shows mean-SEM life span in months for normal-129j, mock-treated GAA KO, and various GL-67-mediated plasmid delivery to GAA KO mice.

## Discussion

Presently there is no cure for PD. ERT using a recombinant human GAA secreted from an overexpressing CHO cell line (Myozyme/Lumizyme) is offered by Sanofi/Genzyme Corp. GT to treat inherited genetic disorders with replication defective retroviruses or adenoviruses containing the normal gene are directed at organs or cell types that can be infected at an efficient rate (e.g., cystic fibrosis transmembrane conductance regulator gene for respiratory epithelium) or for cells that can repopulate organs or the body (e.g., adenosine deaminase deficiency-severe combined immunodeficiency with the adenosine deaminase gene in bone marrow cells or lymphocytes).^55,56^ GT for diseases that affect muscles or organs may be very challenging due to their size or location and current ability to transfect these cells at a reasonable rate. Use of the high-expression CMV immediate early promoter-enhancer element has eliminated some of the expression difficulties.

Studies using DOTMA-DOPE, DDAB-DOPE, or DMRIE-DOPE cationic lipid complexed plasmid DNA containing the CMV promoter administered by intravenous or IP routes in mice demonstrated that 50–150 µg of DNA was widely delivered to tissues with a variety of cell types.^135–137^ After a single injection, they observed the plasmid and its expression for up to 2 months with no toxicity. Tissues containing the plasmid included heart, spleen, kidney, liver, skeletal muscle, lung, bone marrow, stomach, small intestine, colon, thymus, ovary, uterus, macrophages, and T cells. Others have shown that direct instillation of DOTMA/DOPE/DNA complexes into the mouse trachea can mediate pulmonary expression of reporter genes.^151^ Airway epithelium was shown to be the major target of gene transfer, and reporter gene expression lasted at least 4 weeks. No histologic evidence of toxicity was found.

The mechanisms by which cationic lipid-mediated gene transfer and the basis by which cells process and express exogenously delivered plasmid DNA are not well understood. Positively charged lipid head groups have been shown to be necessary in all effective cationic lipid formulations and a polyamine moiety in the head group has recently been reported to enhance DNA expression in certain cell types (compared with lipids containing a head group with a single positive charge). In addition, inclusion of neutral lipids (such as DOPE) within cationic lipid formulations appear to consistently enhance reporter gene expression using cationic lipids.

We chose cationic lipid-mediated gene transfer for a safe and wide transfer to tissues. We utilized GL-67/DOPE/DMPE-Peg5000 (1:2:0.05) that reportedly has high transfection efficiency *in vivo.*^145–149^ It primarily has been used to transfer genes to the airway epithelia by nasal or aerosolized administration with high degrees of success in animals and humans. There has been mild toxicity associated with a dose-dependent inflammatory response characterized by an influx of leukocytes and elevated levels of cytokines IL-6, TNF-α, and IFN-γ.^149^ Most patients exhibited mild airway symptoms, influenza-like symptoms, myalgia, headache, and a temperature roughly 6 h after administration. The cause of most of the inflammatory response was the pDNA and not the lipid, as has been assumed. Zabner et al.^146^ and Yew et al.^149,152^ deleted or mutagenized 270 of 526 CpG dinucleotides in reporter plasmids and were able to significantly reduce the immunostimulatory response *in vitro* and *in vivo*, demonstrating that use of less immunostimulatory vectors could reduce toxicity and increase the therapeutic index of gene transfer.

In this report, we successfully demonstrated that cationic lipid mediated gene transfer *in vivo* in GAA KO mice with the pcDNA3 × human *GAA* cDNA is possible and can correct the enzymatic defect in tissues including the heart, skeletal muscle, and diaphragm. Importantly, this therapy reversed the clinical phenotype of hind and forelimb muscle weakness. In metabolic defects for muscle diseases, it is not clear as to what level of normal enzyme activity must be attained for treatment to improve the clinical phenotype. Residual GAA activity observed in adult patients is 5% to 10% of normal GAA activity where only muscle weakness is observed. We postulate that a 5–10% increase in activity would be sufficient to improved clinical presentation. In this study, treated GAA KO mice showed a modest increase of 7% of normal GAA activity in skeletal muscle and had restoration of muscle activity and strength.

It is important to investigate GT for PD in GAA KO mice. We hypothesized that in treated animals, there would be positive selective pressure to maintain the plasmid in the tissues and cells. Because glycogen has accumulated in the tissues due to the deficiency, any cells that have taken up and expressed the plasmid will be “healthier” by being able to hydrolyze glycogen to glucose, thus providing more energy to survive. In addition, the plasmid most likely was maintained as an episome. However, the positive selection pressure may result in the plasmid integrating stably into the chromosomal DNA. Martin et al.^153^ demonstrated that after intramuscular injection of plasmid DNA, genomic integration could occur. Finally, cationic lipid-mediated gene therapy may be equal or a better alternative than ERT due to less toxicity or immunologic reactions and cost of treatment.

## Data Availability

Data are available from the corresponding author upon reasonable request.
